# Estimation of Soil Salt Content and Organic Matter on Arable Land in the Yellow River Delta by Combining UAV Hyperspectral and Landsat-8 Multispectral Imagery

**DOI:** 10.3390/s22113990

**Published:** 2022-05-25

**Authors:** Mingyue Sun, Qian Li, Xuzi Jiang, Tiantian Ye, Xinju Li, Beibei Niu

**Affiliations:** 1 College of Resources and Environment, Shandong Agricultural University, Taian 271018, China; 2020120469@sdau.edu.cn (M.S.); 2020110362@sdau.edu.cn (X.J.); 2020110355@sdau.edu.cn (T.Y.); lxj0911@126.com (X.L.); 2 Department of Applied Mathematics and Statistics, Stony Brook University, New York, NY 11794, USA; liqian689@gmail.com

**Keywords:** soil salt content, soil organic matter, UAV hyperspectral images, Landsat-8 satellite, Yellow River Delta

## Abstract

Rapid and large-scale estimation of soil salt content (SSC) and organic matter (SOM) using multi-source remote sensing is of great significance for the real-time monitoring of arable land quality. In this study, we simultaneously predicted SSC and SOM on arable land in the Yellow River Delta (YRD), based on ground measurement data, unmanned aerial vehicle (UAV) hyperspectral imagery, and Landsat-8 multispectral imagery. The reflectance averaging method was used to resample UAV hyperspectra to simulate the Landsat-8 OLI data (referred to as fitted multispectra). Correlation analyses and the multiple regression method were used to construct SSC and SOM hyperspectral/fitted multispectral estimation models. Then, the best SSC and SOM fitted multispectral estimation models based on UAV images were applied to a reflectance-corrected Landsat-8 image, and SSC and SOM distributions were obtained for the YRD. The estimation results revealed that moderately salinized arable land accounted for the largest proportion of area in the YRD (48.44%), with the SOM of most arable land (60.31%) at medium or lower levels. A significant negative spatial correlation was detected between SSC and SOM in most regions. This study integrates the advantages of UAV hyperspectral and satellite multispectral data, thereby realizing rapid and accurate estimation of SSC and SOM for a large-scale area, which is of great significance for the targeted improvement of arable land in the YRD.

## 1. Introduction

The Yellow River Delta (YRD) is one of the three largest deltas in China, which is also among the fastest-growing deltas worldwide; the region is rich in natural resources and contains important wetland ecosystems that provide vital habitats for rare and endangered birds [1,2,3,4]. However, the YRD is characterized by shallow groundwater levels and high mineralization due to its exceptional sedimentary environment, which causes soil salt to easily accumulate on the surface [5,6]. High soil salt content (SSC) greatly limits local agricultural productivity by causing soil compaction and reducing soil organic matter content (SOM) [7,8,9]. Soil salinization in the YRD has recently intensified under the influence of driving forces, such as seawater intrusion, climate change, and intensive farming practices, which threaten local food security and ecological security [10,11]. Therefore, there is an urgent need to formulate targeted soil improvement and management measures, which will have far-reaching significance for maintaining the health of the arable land system and regional sustainable development [12,13]. An important step in formulating such measures is to clarify the spatial distribution of SSC and SOM on arable land [14,15].

Remote sensing possesses unique advantages over traditional field surveys in monitoring regional soil salinity and organic matter [16,17,18,19]. As surface reflectance may vary with different content levels of SSC or SOM, a statistical relationship between them can be constructed, which allows the rapid and effective estimation of regional SSC and SOM based on remote sensing data [20,21]. Since the 1970s, a series of satellites (e.g., Landsat, SPOT, and Sentinel) equipped with multispectral sensors have been put into operation. From then, satellite remote sensing images have been widely used in large-scale SSC and SOM estimation for their convenient acquisition, easy processing, and large coverage area [22,23]. Ma et al. used Sentinel-1A and Sentinel-2A data to retrieve the distribution map of soil salinization in the Ogan-Kuqa River Oasis located in the Tarim Basin in Xinjiang, China [24]. Zhai predicted the spatial distribution of SOM in the wetland of Gao’an Research Area and Anyi County of China by combing data from Landsat-8 and GF-1 [16]. However, the estimation accuracy of remotely sensed images is generally lower because satellite image data are easily affected by the atmosphere and clouds [25]. To address this problem, multi-source remote sensing data have gradually been adopted in soil composition estimation studies [26,27]. The combination of near-Earth unmanned aerial vehicle (UAV) multispectral data and satellite remote sensing data is the most widely used approach because UAV images have high resolution and strong anti-interference characteristics, which compensate for the shortcomings of satellite data [18]. Zhu et al. utilized UAV multispectral data, Sentinel-2B remote sensing data, and ground measurement data to construct SSC and SOM estimation models in the coastal saline–alkali land, and produced regional SSC and SOM distribution [28]. Qi et al. inverted SSC on winter wheat areas in the YRD based on UAV multispectral images and Sentinel-2A images [29]. The average ratio adjustment method is a common method for combining UAV and satellite images; it uses the reflectance ratio of corresponding bands with an approximate wavelength range between the UAV and satellite multispectral data to correct the satellite images [30,31]. For example, the average ratio adjustment method has been used to correct Sentinel-2A satellite images to invert SSC in the YRD [32]. However, wavelength range and the central wavelength generally diverge from the corresponding bands of UAV and satellite multispectral data. Therefore, some errors are associated with the estimation result after ratio correction.

Hyperspectral sensors typically collect hundreds of contiguous spectral bands with a narrow bandwidth (approximately 3.5 nm), enabling them to be freely collected into a band combination that matches the spectral range of any band of the satellite multispectral data. Thus, correction accuracy tends to be higher if satellite multispectral data are corrected with UAV hyperspectral data. Recently, UAV hyperspectral images have been used for soil composition estimation within a sampling area. For example, UAV hyperspectral and satellite multispectral data were used to predict SSC distribution data for three sampling areas with different land coverage in the Aksu region, yielding relatively accurate results [33]. However, combined UAV hyperspectral and satellite images are rarely applied in large-scale soil composition estimation, which is worthy of further research.

The main objective of this research is to achieve multi-scale SSC and SOM estimation using a combination of UAV hyperspectral images and a Landsat-8 multispectral image on arable land in the YRD. In this study, the average ratio adjustment method was used to correct the Landsat-8 image to the combination of UAV and satellite data, then the multiple linear regression method was used to construct the SSC and SOM estimation models. The results of this study will provide an important reference for science-based arable-land-use planning, protection of the ecological environment, and soil quality improvement in the YRD [34,35].

## 2. Materials and Methods

### 2.1. Study Area

**Figure 1:**
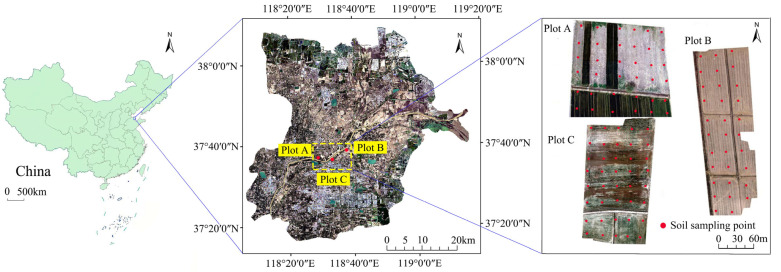
Locations of the study area and test plots.

The study area is within Dongying City, with covers much of the YRD, China (37°16′–38°08′ N, 118°06′–119°18′ E) (Figure 1). The region has a warm temperate, continental monsoon climate with sufficient light and distinct seasons. The terrain is gently sloping, with typical alluvial plain landforms. The main soil types in the study area are coastal saline alkaline soil and fluvo-aquic soils, with high levels of soil salinization [5,36].

Three test plots were established on arable land (Figure 1). Plot A was partly covered with wheat and partly bare land, with an area of nearly 2.77 ha. Plot B was post-rice harvest bare land, and the area was nearly 2.52 ha. Plot C (nearly 3.17 ha) was sparsely covered with wheat. The plots clearly differed in SSC and SOM and were, therefore, used to test the universality of the SSC and SOM estimation model. 

### 2.2. Data Collection and Pre-Processing

#### 2.2.1. Soil Data

Soil samples were collected from the test plots from 12–14 April 2021. To ensure the uniform distribution of sampling points, we placed cards on the ground at 30 m intervals within each plot. A total of 108 sample points were established, including 36, 32, and 40 points in Plots A, B, and C, respectively [37].

The coordinates of each sampling point were recorded using a portable Global Positioning System (GPS) device, and topsoil (depth, 0–10 cm) was collected using the 5-point sampling method around the sampling points.

The soil samples were air dried and ground in the laboratory, and then passed through 0.9 mm and 0.15 mm sieves, sequentially. We selected all samples that had passed through the 0.9 mm sieve to measure SSC using the drying method [38,39]. For all samples (through 0.15 mm sieves), SOM was obtained using dichromate titration [20,40].

#### 2.2.2. UAV Hyperspectral Data

UAV hyperspectral images were obtained simultaneously with soil sampling. A GaiaSky-mini hyperspectral camera was mounted on a Dajiang Matrice 600 Pro UAV (SZ DJI Technology Co., Ltd., Shenzhen, Guangdong Province, China). All UAV flights were carried out between 9:00 and 15:30 under a low wind speed and clear sky. The UAV hovered at a height of 50 m and the ground resolution was set to 2–2.5 cm. The front and side overlaps were, respectively, 75% and 70%. For accurate radiation correction, the ground standard whiteboard image was collected before each UAV flight and the radiation calibration was carried out in the SpecView software. The camera captures 176 bands between 400 and 1000 nm, with an average width of approximately 3.5 nm [41,42]. Image mosaic and orthography of the UAV hyperspectral images were conducted using Agisoft Photoscan software (Agisoft LLC, St. Petersburg, Russia). Additionally, Savitzky–Golay (S–G) filter was applied to the images using ENVI 5.3 software to eliminate the effect of noise on spectral data (ESRI, Redlands, CA, USA) [43].

To eliminate vegetation cover interference in the soil spectrum, linear binary model pixel decomposition was applied to remove vegetation information, and the processed images were used for subsequent analyses [44].

#### 2.2.3. Landsat-8 Satellite Data

The Landsat-8 OLI image covering the study area acquired on 18 April 2021 was obtained from the Landsat-8 Level-1 product provided by the United States Geological Survey (http://earthexplorer.usgs.gov/, accessed on 29 April 2021) [41]. To decrease the effects of atmospheric scattering on image quality, we performed radiation and atmospheric correction [45]. Then the study area with nearly 600,000 ha was clipped from the processed image, and we performed supervised classification using the ENVI v5.3 software. Vegetation spectroscopic information was removed from the Landsat-8 satellite image as described in Section 2.2.2, and the processed image was used for soil estimation in the study area.

### 2.3. Construction and Verification of Estimation Models Based on UAV Images

To achieve SSC and SOM estimation by combining the UAV hyperspectral and satellite data at the scale of the study area, we successively constructed hyperspectral estimation models and fitted multispectral estimation models.

Two and three abnormal samples of SSC and SOM were eliminated on the basis of the Markov distance method combined with soil spectral data. The remaining samples were divided into calibration set and validation set, including 76 calibration sets and 30 validation sets for the SSC samples, and 75 calibration sets and 30 validation sets for the SOM samples.

Pearson correlation analysis was performed to detect the strength of the linear relationship between soil spectral reflectance and measured SSC/SOM, and individual or combined bands with higher correlation coefficients (R) were selected as sensitive parameters for model construction [46,47].

Multiple linear regression methods, which are commonly used in soil composition estimation, were used to construct soil composition hyperspectral and fitted multispectral estimation models, respectively [16,48].

The determination coefficient (*R*^2^), root mean square error (RMSE), and relative percent deviation (RPD) were used to evaluate model accuracy. *R*^2^ reflects the degree of fit of the model, where values approaching 1 indicate better fit, and lower RMSE and higher RPD values indicate better model prediction accuracy [49].

#### 2.3.1. Construction of Hyperspectral Estimation Model

Six mathematical transformations were first performed on the denoised spectral data to better highlight the relationship between SSC/SOM and soil spectral reflectance (Table 1) [50,51]. Then correlation analysis of the measured SSC/SOM and original or transformed spectral values was performed, and sensitive parameters with R > 0.3 were screened out. Finally, these sensitive parameters were used as independent variables, and the SSC or SOM measured values were used as dependent variables to construct hyperspectral estimation models of SSC or SOM using multiple linear regression [52,53].

#### 2.3.2. Construction of Fitted Multispectral Estimation Model

The UAV hyperspectral wavelength range (400–1000 nm) covers the blue, green, red, and near-infrared (NIR) wavelength ranges of the Landsat-8 image. The UAV fitted multispectral data were simulated by averaging the reflectance of the hyperspectral band within the corresponding multispectral band of the Landsat-8 data (reflectance averaging method). The band information is provided in Table 2.

Similarly, the reflectance of the fitted band was mathematically transformed or combined through addition, subtraction, division, logarithmic or reciprocal transformation, or by taking the ratio of addition and division or its reciprocal (Table 3). Fitted individual or combined bands with R > 0.4 were screened as sensitive parameters. Then the SSC and SOM fitted multispectral estimation models were constructed using the linear regression method, and model accuracy was verified as described above.

### 2.4. Landsat-8 Image Reflectance Correction

For the SSC and SOM fitted multispectral estimation models, we corrected the reflectance of the Landsat-8 multispectral image using UAV fitted multispectral data. To ensure the feasibility of correcting the Landsat-8 image based on UAV images, the coordinate range of each sampling point at Landsat-8 image pixels was acquired. Based on this coordinate range, the pixel values (nearly 9000 pixels) of the four bands were extracted at the UAV fitting multispectral images, and we calculated the average reflectance of the UAV fitted multispectral images and Landsat-8 image bands (Blue, Green, Red, and NIR) for all sampling points, and then compared the average reflectance trends of the four bands. Scatter plots for the average reflectance of the corresponding bands were drawn to examine the correlation in reflectance between the UAV and Landsat-8 images.

Average ratio adjustment was performed to normalize the reflectance of the Landsat-8 image [23]. First, the reflectance ratio between the blue bands (Blue/B_B_) of the Landsat-8 image and fitted UAV images was calculated, and then the reflectance correction coefficient of the blue band was calculated as the average of these ratios for all fitted UAV images. The reflectance correction coefficients of all other bands were calculated in the same manner. Finally, the reflectance of each band of the Landsat-8 image was corrected using the corresponding correction coefficient to obtain the corrected satellite image.

### 2.5. SSC and SOM Estimation

SSC and SOM estimation was performed on the scales of the test plots and the study area, respectively. The distribution of SSC/SOM in the test plots was predicted based on the best SSC/SOM hyperspectral estimation models and best SSC/SOM fitted multispectral estimation models based on the UAV images, respectively. Based on the reflectance-corrected Landsat-8 image and the best SSC/SOM fitted multispectral estimation models, SSC and SOM were estimated for the YRD.

The SSC estimation results were classified into grades as follows: non-salinization (0–2 g/kg), mild salinization (2–4 g/kg), moderate salinization (4–6 g/kg), severe salinization (6–10 g/kg), and solonchak (>10 g/kg). The SOM prediction results were classified into grades as follows: very low (0–6 g/kg), low (6–10 g/kg), medium (10–20 g/kg), high (20–30 g/kg), very high (30–40 g/kg), and excessively high (>40 g/kg) [21,54].

## 3. Results and Analysis

### 3.1. SSC and SOM Characteristics

The characteristics of SSC and SOM in soil samples are shown in Table 4. The mean SSC was 5.636 g/kg (0.384–16.193 g/kg) and the mean SOM was 17.081 g/kg (4.001–38.660 g/kg). Both SSC and SOM showed moderate variation among the test plots, according to the coefficients of variation. The distributions of SSC and SOM were similar between the calibration and validation sets and among total soil samples (Table 4).

### 3.2. Construction and Verification of the Hyperspectral Estimation Models

**Figure 2:**
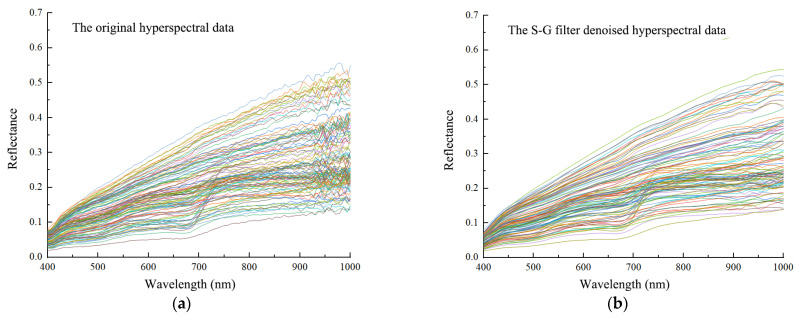
Reflectance of (**a**) original hyperspectral data and (**b**) S–G filter denoised hyperspectral data.

Exploring the characteristic spectral bands of soil can provide a basis for model building. Figure 2 shows the reflectance of original and S–G filter denoised hyperspectral data. The denoised hyperspectral reflectance curves kept the same trends as the original reflectance curves, which highlighted the absorption and reflection features of the reflectance.

**Figure 3:**
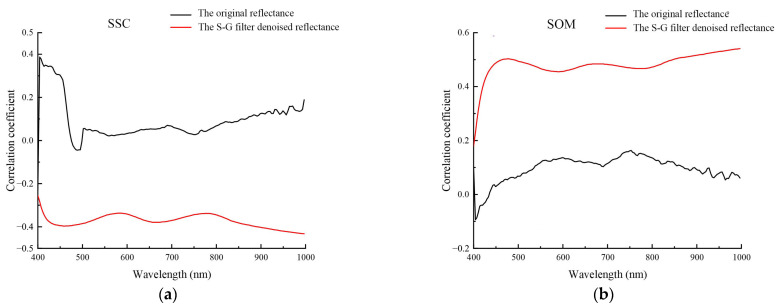
Correlation of original reflectance and S–G filter denoised reflectance for (**a**) SSC and (**b**) SOM.

Compared to the original hyperspectral reflectance, the S–G filter denoised reflectance was more strongly correlated with measured SSC and SOM throughout nearly the entire wavelength range (Figure 3).

**Figure 4:**
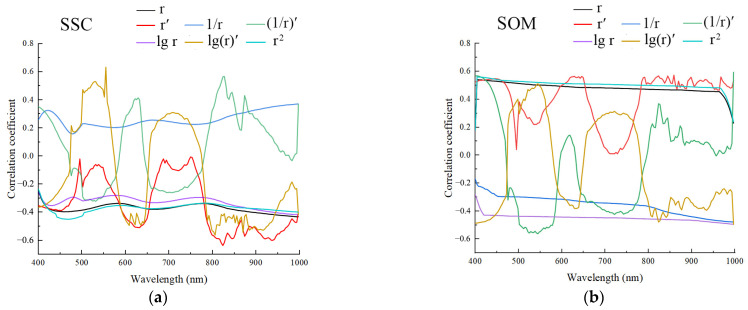
Correlations between hyperspectral reflectance and (**a**) SSC and (**b**) SOM under different mathematical transformations.

To further improve the correlation between measured SSC/SOM and hyperspectral reflectance, we performed six mathematical transformations on the denoised hyperspectral data. The correlation between hyperspectral data under different transformations and measured SSC/SOM is shown in Figure 4. The four transformations (r′, (1/r)′, lg(r)′ and r^2^) effectively improved the correlation between measured SSC and hyperspectral reflectance. The lg(r)′ transformation showed the strongest correlation (|R| = 0.633) at wavelengths of 551.5–554.8 nm, whereas that of the r′ transformation was strongest (|R| = 0.619) at wavelengths of 827.0–830.6 nm; the |R| of (1/r)′ and r^2^ transformation was 0.568 and 0.450, respectively. Among all transformations, (1/r)′ had the most significant improvement in the correlation between SOM and reflectance, reaching a peak (|R| = 0.594) at wavelengths of 996.9–1000 nm. Moreover, r′ and r^2^ also improved the correlation, and the |R| of r′ and r^2^ transformation was 0.569 and 0.560, respectively.

The correlations under the lg(1/r) and 1/r transformations were significantly weaker than those of the non-transformed data (r); therefore, we used only the sensitive parameters identified using the r, r′, (1/r)′, lg(r)′ and r^2^ transformations to construct the SSC and SOM hyperspectral estimation models (Table 5). Among the five SSC hyperspectral estimation models, F(lg(r)′) had the highest accuracy; the calibration *R*^2^ and RMSE values were 0.779 and 1.676 g/kg, respectively, and the validation *R*^2^, RMSE, and RPD values were 0.761, 1.799 g/kg, and 2.004, respectively. The SOM hyperspectral estimation model constructed using the sensitive parameters under the (1/r)’ transformation had the highest accuracy; the calibration *R*^2^ and RMSE values were 0.763 and 4.139 g/kg, respectively, and the validation *R*^2^, RMSE, and RPD values were 0.757, 4.553 g/kg, and 2.001, respectively.

### 3.3. Construction and Verification of the Fitted Multispectral Estimation Model

Table 6 shows the sensitive SSC and SOM parameters (|R| > 0.4) filtered from the fitted multispectral bands and their transformations. Next, the sensitive SSC and SOM parameters were divided into two groups each. Groups S1 and M1 included 25 and 23 parameters, respectively, with |R| > 0.4, and groups S2 and M2 included 8 and 5 parameters, respectively, with |R| > 0.55.

The fitted multispectral models (SSC1, SSC2, SOM1, and SOM2) were constructed using the multiple linear regression method based on the four groups (S1, S2, M1, and M2) (Table 7). Only five sensitive parameters were retained in each model. For the SSC1 model, the calibration *R*^2^ and RMSE values were 0.691 and 1.938 g/kg, respectively, and the validation *R*^2^, RMSE, and RPD values were 0.676, 2.202 g/kg, and 1.743, respectively. For the SOM1 model, the calibration *R*^2^ and RMSE values were 0.684 and 5.105 g/kg, respectively, and the validation *R*^2^, RMSE, and RPD values were 0.663, 5.263 g/kg, and 1.691, respectively. The SSC1 and SOM1 models, which were constructed using the group S1 and M1 parameters, respectively, as independent variables, showed greater precision than the SSC2 and SOM2 models, which were constructed using group S2 and M2 parameters, respectively. Thus, the relevance of the sensitive parameters can lead to missing information in the model, reducing its accuracy.

**Figure 5:**
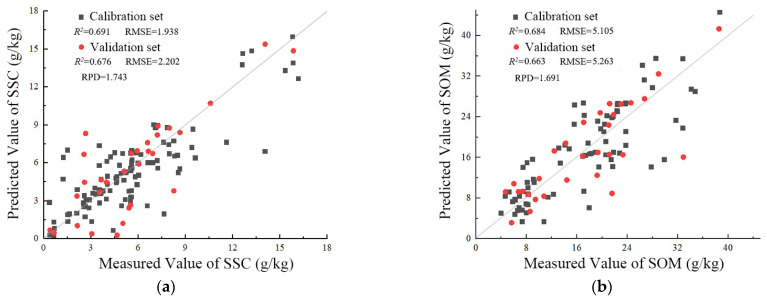
Scatter diagram of the (**a**) SSC1 and (**b**) SOM1 model.

Moreover, Figure 5 showed that the predicted values of the SSC and SOM of SSC1 and SOM1 models were in good agreement with their measured values. This proves that models SSC1 and SOM1 have relatively stable prediction ability. Therefore, models SSC1 and SOM1 were selected as the optimal fitted multispectral estimation models for SSC and SOM, respectively, and were used in the subsequent multi-scale map.

### 3.4. SSC and SOM Estimation in the Test Plot

**Figure 6:**
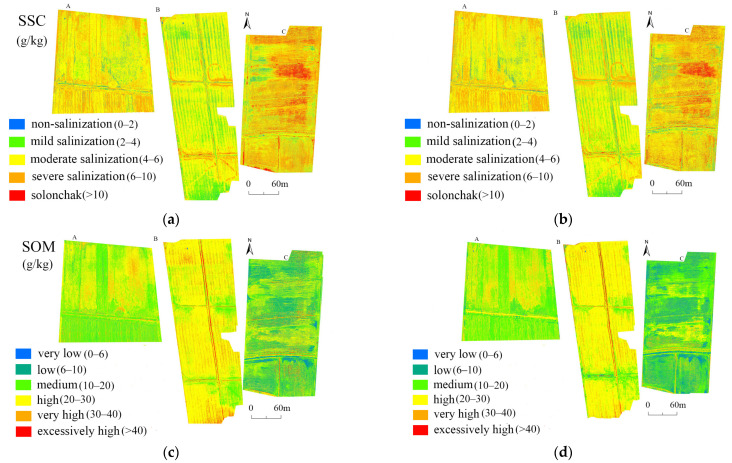
Distribution of (**a**) SSC and (**c**) SOM based on the hyperspectral estimation model and that of (**b**) SSC and (**d**) SOM based on the fitted multispectral estimation model in the three test plots.

Figure 6 shows the distributions of SSC and SOM in the three test plots based on the hyperspectral estimation model and the fitted multispectral estimation model. The proportions of area associated with each SSC and SOM grade are listed in Table 8.

The distribution of SSC and SOM in the three plots exhibited high spatial heterogeneity. In Plot A, the range of SSC was approximately 4–10 g/kg, with moderate or severe soil salinization. In Plot B, SSC was mainly 2–4 g/kg, with low soil salinization. Plot C showed higher degrees of salinization than the other plots, with greater SSC in the east than in the west. The degree of soil salinization among the plots occurred as follows, in descending order: C > A > B. In Plot A, the range of SOM was 0–40 g/kg (medium), with higher values near the center of the plot than near its boundaries. In Plot B, SOM was 20–30 g/kg (high) in >76.53% of the total area of arable land. In Plot C, SOM ranged mainly from 6 to 10 g/kg (low) throughout the entire region. Throughout the plots, SSC and SOM showed a clear negative spatial correlation [55].

Overall, the spatial distributions of SSC and SOM based on the hyperspectral models and the fitted multispectral models for each plot were roughly identical, which demonstrates that the best SSC and SOM fitted multispectral models (SSC1 and SOM1 models) can be used for large-scale prediction.

### 3.5. Landsat-8 Image Reflectance Correction

**Figure 7:**
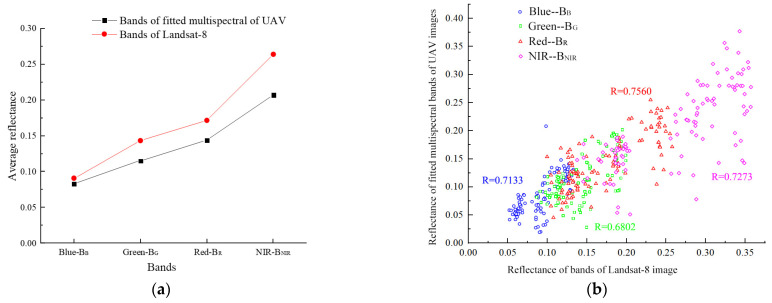
Comparison (**a**) and scatter plot (**b**) of surface reflectance of the fitted multispectral bands of the UAV images and bands of the Landsat-8 image.

A comparison of reflectance between the fitted multispectral bands (B_B_, B_G_, B_R_, and B_NIR_) of the UAV images and the Landsat-8 satellite image bands (Blue, Green, Red, and NIR) is shown in Figure 7. As seen in Figure 7a, the average reflectance of the four Landsat-8 image bands was higher than for the fitted UAV image bands; however, their trends were consistent. Furthermore, the reflectance correlation coefficient between the Landsat-8 image Red band and the corresponding fitted UAV band (B_R_) was 0.756, showing good correlation. The reflectance of the NIR and Blue bands of the Landsat-8 image was moderately correlated with those of the corresponding fitted UAV multispectral bands (both, R > 0.7). The correlation between the Green and B_G_ bands was relatively low (0.6802), although still within the acceptable range (Figure 7b) [28]. Therefore, UAV fitted multispectral images could be fused with Landsat-8 image for large-scale SSC and SOM prediction.

The reflectance correction coefficient of each band was calculated as the average reflectance ratio of the Landsat-8 image to the fitted UAV images (Table 9). Then, the reflectance of four bands of the Landsat-8 images was divided by the correction coefficient of the corresponding band to obtain the corrected Landsat-8 image.

### 3.6. SSC and SOM Estimation in the Study Area

**Figure 8:**
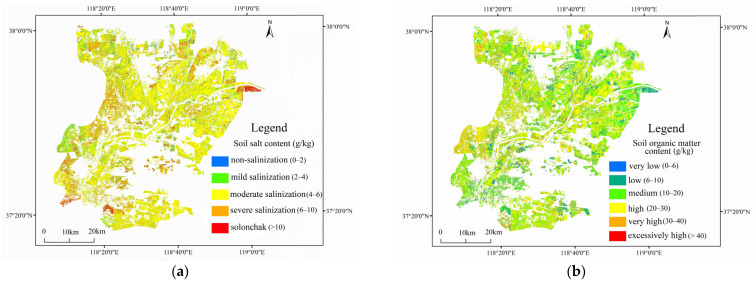
Spatial distribution of retrieved (**a**) SSC and (**b**) SOM values in the study area.

Figure 8 shows the SSC and SOM prediction results for the study area based on the best fitted multispectral estimation models and the corrected Landsat-8 image. Table 10 summarizes the area coverage of each SSC and SOM grade.

The moderate salinization class (SSC = 4–6 g/kg) accounted for the largest area (48.44% of the total area of arable land), widely distributed throughout the YRD. By contrast, severe salinization (SSC = 6–10 g/kg) was concentrated in southern Hekou District, western Lijin County, and northern Dongying District. Areas classified as solonchak (SSC > 10 g/kg) represented 6.40% of the study area; these were mainly concentrated in eastern regions, due to low groundwater levels [56,57]. Overall, the degree of soil salinization gradually increased from high to low terrain and from upstream to downstream [21].

Areas with medium SOM (10–20 g/kg) accounted for 43.11% of the study area, distributed to varying degrees throughout the study region. Areas with high SOM (20–30 g/kg) were mainly distributed in northern Kenli District, accounting for 24.53%. Few areas had very high SOM (30–40 g/kg); these were mainly distributed along the Yellow River and the ancient Yellow River channel, where irrigation water sources are abundant and contribute to the accumulation of organic matter [3]. Low SOM (<10 g/kg) was associated with high salinization in this area.

## 4. Discussion

Vegetation cover interferes with soil spectral reflectance, which causes discrepancies between spectral information in the collected images and the actual surface soil reflectance. To improve the accuracy of spectral prediction results, in this study, we obtained spectral images acquired in spring, when vegetation coverage is low, and used the linear binary model pixel decomposition method to remove vegetation information from the images to further eliminate interference [21,44].

UAV multispectral images and satellite images have been widely used for combined satellite–UAV estimation. However, there are usually deviations in the wavelength range and central wavelength between corresponding bands of the UAV and satellite multispectral images, which are associated with errors in average ratio adjustments based on the two datasets, and, in turn, affect prediction accuracy [21,54]. Hyperspectral sensors typically collect hundreds of contiguous spectral bands with narrow bandwidths (approximately 3.5 nm), which allow it to be freely collected into a band combination that matches the wavelength range of any satellite multispectral band. Thus, the use of UAV hyperspectral data in correcting satellite multispectral data leads to higher correction accuracy [42].

In this study, the best SSC and SOM hyperspectral estimation models showed higher precision than the corresponding best fitted multispectral estimation models. This result may be explained by the loss of spectral information when the fitted multispectral data (4 bands) are constructed using hyperspectral data (176 bands), which reduces the correlation between spectral reflectance and measured SSC and SOM values. Following mathematical transformation and combination, although the correlation and spectral information richness of the fitted multispectral data were improved, they remained inferior to the hyperspectral data after appropriate mathematical transformation. Therefore, enriching the fitted multispectral information and improving the correlation will be a focus of future research.

In this study, the combination of UAV hyperspectral data and satellite images improved SSC and SOM prediction accuracy to some extent; however, our study had limitations. First, we estimated only soil composition data in spring, and the content of soil components varies seasonally, resulting in changes in the relationships between soil components and the image spectrum. Therefore, the best estimation model obtained in this study would not be applicable in other seasons; further research is required to extend the models seasonally. In addition, we used only linear regression to construct the estimation models; however, many studies have confirmed that machine-learning-based modelling methods, such as support vector machine, back propagation neural network, and random forest algorithms, also perform well in soil prediction [24,58,59]. These methods will be evaluated in subsequent research.

Clarifying the spatial distributions of SSC and SOM on arable land is key to formulating effective soil improvement and management measures. In this study, SSC and SOM were negatively spatially correlated on arable land in the YRD. Areas with high SSC and low SOM were mainly distributed in western Lijin County, eastern Kenli County, southern Hekou District, and northern Dongying District. In recent years, intensive farming and excessive inorganic fertilizer application have been frequently conducted to increase crop yields in the YRD; these unsustainable practices increase the SSC and accelerate the loss of SOM [31]. To alleviate exacerbated soil salinization and accelerate SOM accumulation in the YRD, particularly in the regions identified in this study, various measures, such as rational cultivation, scientific irrigation, and adjustment of crop planting structure, require implementation to improve arable land quality and develop the agricultural economy.

## 5. Conclusions

This paper presents an effective method to map large-scale SSC and SOM in arable land in the YRD by integrating the advantages of UAV hyperspectral data (accurate and efficient) and the satellite data (wide range). The results indicate that lg(*r*)′ and (1/*r*)′ are the most effective mathematical transformations to improve the correlation of reflectance with SSC and SOM, respectively. Correspondingly, the models F(lg(r)′) and F(1/r)′), constructed with sensitive parameters that underwent these two transformations, showed the best SSC and SOM estimation effect, with *R*^2^ of 0.779 and 0.761, respectively. The best SSC and SOM fitted multispectral estimation models (SSC1/SOM1) were constructed using sensitive parameters with |R| > 0.4. The obtained SSC and SOM distribution showed that soil salinization exists in most areas in the YRD. Areas classified as solonchak represented 6.40% of the total area of arable land, with moderately and severely salinized soil covering 76.35%. Low or medium SOM accounted for 60.31% of the total arable land in the YRD. SSC and SOM were negatively spatially correlated. These results will provide a scientific reference for the targeted improvement of arable land quality and ecological environment protection in the YRD.

## Figures and Tables

**Table 1:** Mathematical transformation form of hyperspectral reflectance.

Transformation Form	Symbol
Untransformed reflectance	r
First-order differential	r′
Reciprocal	1/r
First-order differential of reciprocal	(1/r)′
Logarithmic	lg r
First-order differential of logarithm	lg(r)′
Square	r^2^

**Table 2:** Band information for Landsat-8 satellite multispectral data and fitted unmanned aerial vehicle (UAV) multispectral data.

Satellite Bands	Landsat-8 Data		Fitted Multispectral Bands	UAV Data	
	Wavelength Coverage (nm)	Central Wavelength (nm)		Wavelength Coverage (nm)	Central Wavelength (nm)
Blue (B)	450–515	482.5	B_B_	449.4–515.0	482
Green (G)	525–600	562.5	B_G_	524.9–598.5	561.5
Red (R)	630–680	655	B_R_	632.3–680.2	656
Near-infrared (NIR)	845–885	865	B_NIR_	844.8–884.2	866.2

**Table 3:** Mathematical transformations of fitted multispectral data.

Transformation Form	Symbol *
Addition	B_i_ + B_j_
Subtraction	B_i_ − B_j_
Division	B_i_/B_j_
Logarithmic	lg(B_i_)
Reciprocal	1/B_i_
Ratio of addition and division	(B_i_ + B_j_)/(B_i_ − B_j_)
Ratio of division and addition	(B_i_ − B_j_)/(B_i_ + B_j_)

* B_i_ and B_j_ are the reflectance of bands i and j, respectively, where i and j are defined as B, G, R, or NIR bands, and i ≠ j.

**Table 4:** Soil salt content (SSC) and soil organic matter (SOM) characteristics among soil samples.

	Sample Type	Sample Size	Minimum (g/kg)	Maximum (g/kg)	Mean (g/kg)	Standard Deviation (g/kg)	Coefficient of Variation
SSC	Total	106	0.384	16.193	5.636	3.415	0.605
	Calibration set	76	0.384	16.193	5.614	3.407	0.607
	Validation set	30	0.406	15.855	5.692	3.435	0.603
SOM	Total	105	4.001	38.660	17.081	8.461	0.495
	Calibration set	75	4.001	38.660	17.038	8.479	0.498
	Validation set	30	4.692	38.531	17.191	8.413	0.489

**Table 5:** SSC and SOM hyperspectral estimation models.

Model	SSC Dataset					SOM Dataset				
	Calibration Set		Validation Set			Calibration Set		Validation Set		
	*R* ^2^	RMSE (g/kg)	*R* ^2^	RMSE (g/kg)	RPD	*R* ^2^	RMSE (g/kg)	*R* ^2^	RMSE (g/kg)	RPD
F(r)	0.671	2.035	0.616	2.268	1.503	0.732	4.798	0.711	4.854	1.791
F(r)′	0.767	1.679	0.744	1.821	1.924	0.747	4.443	0.733	5.175	1.934
F(1/r)′	0.726	1.906	0.739	1.777	1.537	0.763	4.138	0.757	4.553	2.001
F((lg r)′)	0.779	1.676	0.761	1.799	2.004	0.693	5.134	0.696	4.984	1.711
F(r^2^)	0.692	2.059	0.715	1.973	1.713	0.744	4.702	0.736	4.740	1.935

**Table 6:** Sensitive parameters used to construct the SSC and SOM fitted multispectral estimation models.

|R|	SSC Fitted Multispectral Estimation Models		|R|	SOM Fitted Multispectral Estimation Models	
	Group S1 (|R| > 0.40)	Group S2 (|R| > 0.55)		Group M1 (|R| > 0.40)	Group M2 (|R| > 0.55)
0.45	B_B_		0.50	B_B_	
0.49	B_G_		0.46	B_G_	
0.45	B_R_		0.48	B_R_	
0.51	B_NIR_		0.51	B_NIR_	
0.45	B_B_ + B_NIR_		0.48	lg(B_NIR_)	
0.62	B_R_ + B_NIR_	B_R_ + B_NIR_	0.48	B_B_ + B_G_	
0.45	B_B_ − B_R_		0.49	B_B_ + B_R_	
0.42	B_G_ − B_NIR_		0.56	B_B_ + B_NIR_	
0.58	B_G_/B_R_	B_G_/B_R_	0.47	B_G_ + B_R_	
0.46	(B_B_ + B_G_)/(B_B_ − B_G_)		0.50	B_G_ + B_NIR_	
0.48	(B_B_ + B_R_)/(B_B_ − B_G_)		0.44	B_R_ + B_NIR_	
0.64	(B_B_ + B_NIR_)/(B_B_ − B_G_)	(B_B_ + B_NIR_)/(B_B_ − B_G_)	0.48	B_B_ − B_NIR_	
0.60	(B_G_ + B_R_)/(B_B_ − B_G_)	(B_G_ + B_R_)/(B_B_ − B_G_)	0.53	B_G_ − B_R_	
0.44	(B_G_ + Bnir)/(B_B_ − B_G_)		0.51	B_G_ − B_NIR_	
0.60	(B_R_ + B_NIR_)/(B_B_ − B_G_)	(B_R_ + B_NIR_)/(B_B_ − B_G_)	0.46	B_G_/B_R_	
0.44	(B_R_ + B_NIR_)/(B_G_ − B_R_)		0.42	(B_B_ + B_G_)/(B_B_ − B_G_)	
0.52	(B_G_ − B_R_)/(B_B_ + B_G_)		0.56	(B_B_ + B_R_)/(B_B_ − B_G_)	(B_B_ + B_R_)/(B_B_ − B_G_)
0.45	(B_G_ − B_R_)/(B_B_ + B_R_)		0.61	(B_B_ + B_NIR_)/(B_B_ − B_G_)	(B_B_ + B_NIR_)/(B_B_ − B_G_)
0.62	(B_B_ − B_G_)/(B_B_ + B_NIR_)	(B_B_ − B_G_)/(B_B_ + B_NIR_)	0.56	(B_G_ + B_R_)/(B_B_ − B_G_)	(B_G_ + B_R_)/(B_B_ − B_G_)
0.56	(B_G_ − B_R_)/(B_B_ + B_NIR_)	(B_G_ − B_R_)/(B_B_ + B_NIR_)	0.61	(B_G_ + B_NIR_)/(B_B_ − B_G_)	(B_G_ + B_NIR_)/(B_B_ − B_G_)
0.51	(B_G_ − B_R_)/(B_G_ + B_R_)		0.46	(B_B_ − B_G_)/(B_G_ + B_R_)	
0.48	(B_G_ − B_R_)/(B_G_ + B_NIR_)		0.47	(B_G_ − B_R_)/(B_G_ + B_R_)	
0.56	(B_B_ − B_G_)/(B_R_ + B_NIR_)	(B_B_ − B_G_)/(B_R_ + B_NIR_)	0.57	(B_B_ − B_G_)/(B_R_ + B_NIR_)	(B_B_ − B_G_)/(B_R_ + B_NIR_)
0.45	(B_G_ − B_R_)/(B_R_ + B_NIR_)				
0.41	(B_G_ − B_NIR_)/(B_R_ + B_NIR_)				

**Table 7:** SSC and SOM fitted multispectral estimation models.

Model	Parameters	Formula	Calibration Set		Validation Set		
			*R* ^2^	RMSE (g/kg)	*R* ^2^	RMSE (g/kg)	RPD
SSC1	Group S1 (|R| > 0.40)	Y = 44.637 − 81.464 × B_NIR_ − 11.690 × (B_B_ + B_NIR_)/(B_B_ − B_G_) − 5.56 × (B_G_ + B_R_)/(B_B_ − B_G_) + 54.909 × (B_B_ − B_G_)/(B_B_ + B_NIR_) + 34.665 × (B_B_ − B_G_)/(B_R_ + B_NIR_)	0.691	1.938	0.676	2.202	1.743
SSC2	Group S2 (|R| > 0.55)	Y = 57.412 − 16.666 × (B_G_/B_R_) − 10.153 × (B_B_ + B_NIR_)/(B_B_ − B_G_) − 1.285 × (B_G_ + B_R_)/(B_B_ − B_G_) + 13.275 × (B_R_ + B_NIR_)/(B_B_ − B_G_) + 63.189 × (B_B_ − B_G_)/(B_B_ + B_NIR_)	0.659	2.180	0.655	2.240	1.676
SOM1	Group M1 (|R| > 0.40)	Y = − 109.761 + 61.143 × (B_G_ + B_NIR_) + 17.294 × (B_B_ + B_R_)/(B_B_ − B_G_) − 13.642 × (B_B_ + B_NIR_)/(B_B_ − B_G_) − 904.36 × (B_B_ − B_G_)/(B_B_ + B_NIR_) + 887.385 × (B_B_ − B_G_)/(B_R_ +B_NIR_)	0.684	5.105	0.663	5.263	1.691
SOM2	Group M2 (|R| > 0.55)	Y = − 65.888 + 4.266 × (B_B_ + B_R_)/(B_B_ − B_G_) − 27.725 × (B_B_ + B_NIR_)/(B_B_ − B_G_) + 11.55 × (B_G_ + B_NIR_)/(B_B_ − B_G_) − 1236.432 × (B_G_ + B_R_)/(B_B_ − B_G_) + 1398.119 × (B_B_ − B_G_)/(B_R_ + B_NIR_)	0.649	5.340	0.656	5.325	1.655

**Table 8:** Areal distribution of SSC and SOM in the three test plots.

Plot	SSC Grade (g/kg)	Area (%)		SOM Grade (g/kg)	Area (%)	
		Hyperspectral Estimation Model	Fitted Multispectral Estimation Model		Hyperspectral Estimation Model	Fitted Multispectral Estimation Model
A	0–2	1.76	3.07	0–6	0.05	0.17
	2–4	7.13	4.31	6–10	0.20	0.37
	4–6	54.25	50.29	10–20	70.58	78.74
	6–10	36.86	42.33	20–30	22.34	13.31
	>10	0	0	30–40	6.83	7.41
				≥40	0	0
B	0–2	0.12	0.09	0–6	0.02	0.41
	2–4	45.31	45.27	6–10	0.03	0.43
	4–6	48.13	44.62	10–20	14.93	15.15
	6–10	6.26	9.93	20–30	76.61	76.53
	≥10	0.18	0.09	30–40	3.29	3.93
				≥40	5.12	3.55
C	0–2	0.27	0.23	0–6	7.04	8.91
	2–4	4.58	3.71	6–10	51.14	45.36
	4–6	16.91	24.01	10–20	24.15	27.98
	6–10	59.43	57.22	20–30	16.44	15.98
	≥10	18.81	14.83	30–40	0.64	1.73
				≥40	0.59	0.04

**Table 9:** Reflectance correction coefficient of the Landsat-8 satellite image.

Band	Blue	Green	Red	NIR
Reflectance correction coefficient	1.09	1.25	1.19	1.27

**Table 10:** Area (%) of soil showing different grades of SSC and SOM in the study area.

SSC Grade (g/kg)	Area (km^2^)	Percentage (%)	SOM Grade (g/kg)	Area (km^2^)	Percentage (%)
0–2	17.06	0.80	0–6	91.43	4.06
2–4	354.25	16.45	6–10	296.32	13.14
4–6	1043.08	48.44	10–20	971.90	43.11
6–10	600.93	27.91	20–30	553.01	24.53
>10	137.85	6.40	30–40	329.95	14.63
			>40	11.99	0.53

## Data Availability

Not applicable.
